# Research progress on the mechanisms of endogenous neural stem cell differentiation in spinal cord injury repair

**DOI:** 10.3389/fncel.2025.1592297

**Published:** 2025-06-18

**Authors:** Tianwei Wang, Qing Han, Shi Lv, Li-ping Zhang, Hengrui Li, Jian Liu, Jinyi Kuang, Bao-liang Sun, Jing-yi Sun

**Affiliations:** ^1^Department of Neurology, The Second Affiliated Hospital, Shandong First Medical University and Shandong Academy of Medical Sciences, Taian, China; ^2^Institute of Brain Science and Brain-inspired Research, Shandong First Medical University and Shandong Academy of Medical Sciences, Jinan, China; ^3^School of Traditional Chinese Medicine, Shandong University of Traditional Chinese Medicine, Jinan, China; ^4^Department of Neurology, Shandong Second Provincial General Hospital, Jinan, Shandong, China; ^5^Department of Spinal Surgery, Shandong Provincial Hospital Affiliated to Shandong First Medical University, Jinan, China

**Keywords:** endogenous neural stem cells, spinal cord injury, neural differentiation mechanisms, signaling pathways, biomaterials, neural regeneration

## Abstract

Spinal cord injury (SCI) is a devastating condition with limited self-repair capacity, resulting in long-term disabilities. Endogenous neural stem cells (eNSCs), which are present in the adult central nervous system (CNS), hold significant potential for repairing neural damage following SCI. These cells can proliferate, migrate to the injury site, and differentiate into various neural cell types, including neurons and glial cells. However, after SCI, eNSCs predominantly differentiate into astrocytes, with minimal neuronal differentiation, thereby hindering effective neural regeneration. This review summarizes the key mechanisms underlying the differentiation of eNSCs into neurons, focusing on the molecular signaling pathways that regulate their fate, including the Notch, Wnt/β-catenin, Sonic Hedgehog, and PI3K/Akt pathways. It also discusses the microenvironment’s role, including factors such as hypoxia, extracellular matrix components, and inflammatory cytokines, which influence eNSCs differentiation. The review also highlights potential therapeutic strategies to enhance eNSCs differentiation into neurons, including biomaterials and multimodal approaches that combine pharmacological, physical, and tissue engineering techniques. Despite progress in understanding eNSCs biology and signaling mechanisms, challenges remain in optimizing therapeutic strategies for SCI repair. Future research should focus on overcoming these limitations, emphasizing refining treatment timing, drug delivery systems, and the development of personalized therapies to promote effective neural regeneration and functional recovery after SCI.

## 1 Introduction

SCI is a severe neurological condition that leads to the loss of motor, autonomic, and sensory functions below the injury site (Li et al., 2024a). SCI disrupts the CNS, interrupts neural circuits, and triggers a cascade of functional deficits. It often results in irreversible sequelae, including paralysis, respiratory complications, and bladder dysfunction, primarily due to the CNS’s limited capacity for self-repair (Eckert and Martin, 2017; Li et al., 2023). Following SCI diagnosis, immediate interventions such as hemodynamic monitoring in intensive care, early decompression surgery, blood pressure support, and methylprednisolone administration are crucial. Although these measures can effectively prevent further deterioration of neurological function and reduce complications, the chances of functional recovery remain low (Ahuja et al., 2017).

According to epidemiological studies, approximately 760,000 new cases of SCI occur globally each year, with an annual incidence rate of 10.5 cases per 100,000 individuals (Kumar et al., 2018). In China, the standardized prevalence in 2010 was 569.7 cases per million people (Jiang et al., 2021a). In the United States, approximately 17,700 new cases are reported annually, while in European countries, the annual incidence varies widely, ranging from 5.5 to 195.4 cases per million population (Aschauer-Wallner et al., 2021). With population aging and the increasing exposure to high-risk factors such as traffic accidents and falls, the incidence of SCI has shown a rising trend yearly, imposing a persistent and substantial burden on patients, families, and public healthcare systems (Collaborators, 2019).

The functional recovery of SCI remains challenging. This is mainly due to the onset of secondary injury mechanisms, such as vascular damage, excitotoxicity, and ionic imbalance, which lead to neuronal death, axonal disruption, and ultimately permanent neurological dysfunction by damaging neural circuits (Assinck et al., 2017; Eckert and Martin, 2017; Alizadeh et al., 2019; Hu et al., 2023). The eNSCs are undifferentiated, multipotent cells within the adult CNS, possessing self-renewal potential and the ability to differentiate into neurons, glial fibrillary acidic protein-positive astrocytes (GFAP^+^), and oligodendrocytes. Promoting their differentiation into neurons is critical for replacing lost neurons and repairing damaged neural circuits. Following SCI, eNSCs are activated, proliferate, and migrate toward the injury site. Approximately 95% of these cells differentiate into astrocytes, with 53% originating from activated eNSCs and the remaining 47% deriving from the proliferation of resident GFAP^+^ cells (Barnabe-Heider et al., 2010; Li et al., 2019). This process is a key factor contributing to the failure of neuronal regeneration. Therefore, exploring methods to activate and promote the differentiation of eNSCs into neurons has become a promising non-invasive therapeutic strategy for SCI (Liu and Chen, 2019; Gilbert et al., 2022; Li et al., 2023). This review aims to summarize and analyze the mechanisms underlying the differentiation of eNSCs, providing a comprehensive framework that elucidates their potential role in SCI repair.

## 2 Pathophysiology of SCI

**Figure 1:**
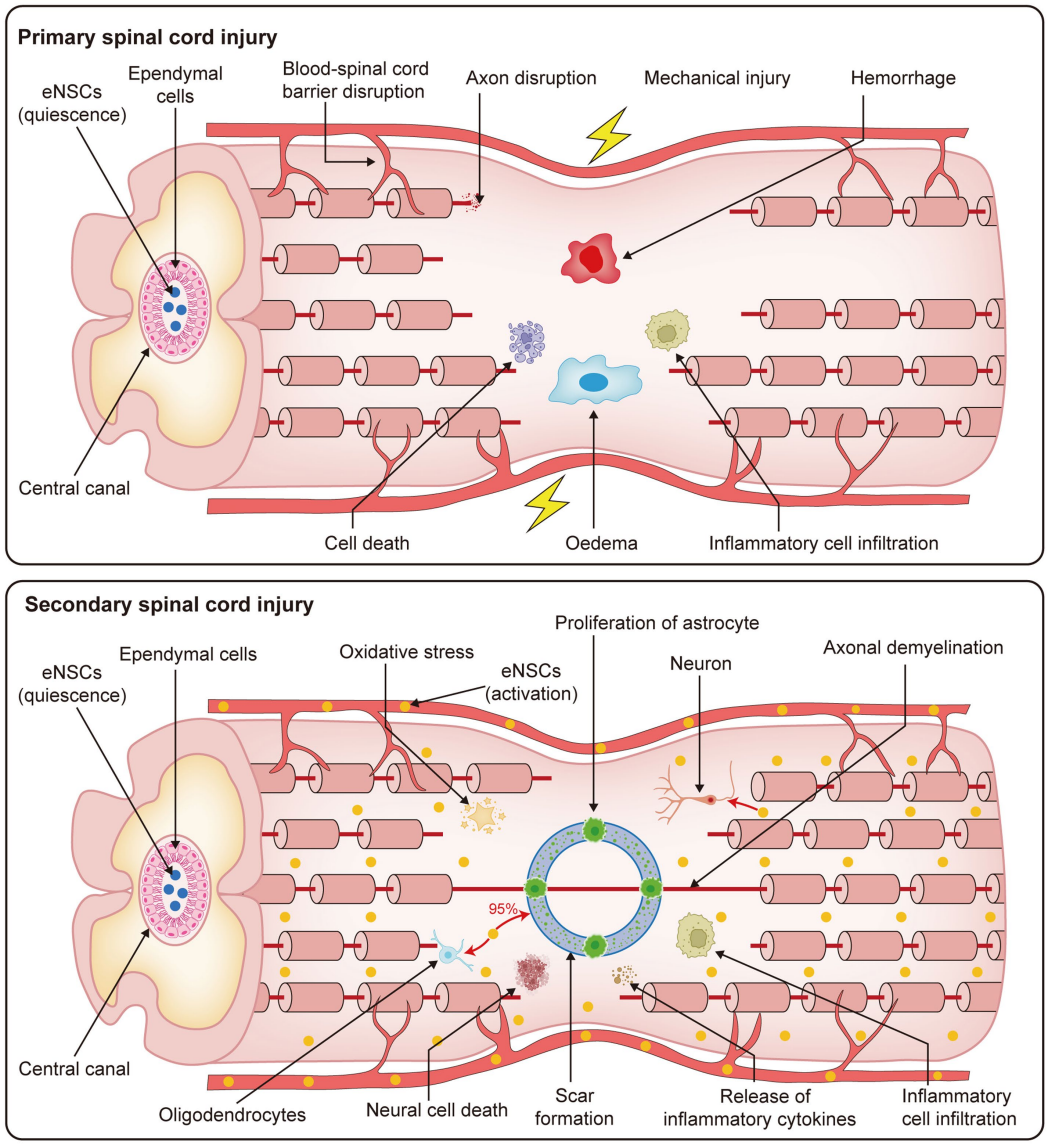
Pathophysiological processes of primary and secondary SCI. This figure presents the progression of SCI, which is divided into two stages: primary and secondary injury. The primary phase occurs immediately following the traumatic event, with rapid disruption of the spinal cord microenvironment. Key events include blood-spinal cord barrier disruption, axonal disruption, mechanical injury, hemorrhage, cell death, edema, and inflammatory cell infiltration. These pathological changes result in severe and irreversible damage to the spinal cord tissue. The secondary injury phase follows, which is characterized by ongoing cellular and molecular responses to the initial trauma. This stage involves oxidative stress, proliferation of astrocytes, axonal demyelination, neural cell death, scar formation, release of inflammatory cytokines, and further inflammatory cell infiltration. Together, these processes contribute to the prolonged and worsening damage to the spinal cord, exacerbating the injury and hindering potential recovery mechanisms.

SCI is a significant form of traumatic injury to the CNS, characterized by high disability and mortality rates. The pathological progression of SCI can be divided into primary and secondary stages, both of which significantly disrupt the spinal cord microenvironment (Li et al., 2023; Shen and Cai, 2023). Mechanical trauma triggers the primary injury phase, characterized by rapid pathological events such as cellular necrosis, axonal membrane rupture, and blood-spinal cord barrier disruption (Ahuja et al., 2017; Li et al., 2024a). These events typically occur within minutes of injury, accompanied by bone fragment displacement and spinal ligament rupture (Choo et al., 2007). Secondary injury follows the primary insult and results from cellular and biological responses to the initial damage. This phase involves multiple systems, including the immune, nervous, and vascular systems, which contribute to further pathological changes such as hemorrhage, ischemia, oxidative stress, inflammation, neuronal cell death, demyelination, and scar formation (Anjum et al., 2020; Hu et al., 2023). These processes exacerbate tissue damage, including glial proliferation, glial scarring, inflammatory responses, and other reversible pathological alterations (Katoh et al., 2019). The progression of secondary injury leads to profound disruption of the spinal cord microenvironment, characterized by dynamic changes at the molecular, cellular, and systemic levels (Fan et al., 2018a). These changes include a reduction in beneficial repair factors and an increase in harmful inflammatory mediators. Certain factors, such as elevated soluble factor levels, hypoxic conditions, and immune responses, can stimulate the activation of eNSCs, promote their proliferation, and facilitate their differentiation (Yagita et al., 2002; Perez Estrada et al., 2014; Covacu and Brundin, 2017; Li et al., 2023). During the primary phase of SCI, eNSCs predominantly remain quiescent. They are localized around the ependymal region of the central canal, which is considered the principal niche of eNSCs in the adult spinal cord (Moreno-Manzano, 2020). Under normal physiological conditions, these cells exhibit a very low proliferation rate and remain undifferentiated. During the secondary phase of injury, quiescent eNSCs become activated in response to factors such as inflammation, hypoxia, reactive oxygen species generation, and the release of cytokines (Hachem et al., 2020). This activation is characterized by enhanced proliferative capacity and a progressive loss of their quiescent phenotype (Finkel et al., 2021; Figure 1). Therefore, understanding the mechanisms underlying the activation of eNSCs following SCI, especially optimizing the microenvironment or implementing targeted interventions to guide their differentiation into functional cell types (such as neurons or oligodendrocytes), is crucial not only for revealing the fundamental processes involved in spinal cord regeneration but also for advancing the development of more effective and targeted therapeutic strategies.

## 3. Characteristics of neural stem cells (NSCs) and their response after spinal cord injury

3

### 3.1 Basic properties of NSCs

NSCs are pluripotent, self-renewing cells that can differentiate into neurons, astrocytes, and oligodendrocytes (Li et al., 2023). These pluripotent cells are essential for nervous system development, maintenance, and repair (Weiss et al., 1996). The functional characteristics of NSCs are tightly regulated by intrinsic and extrinsic signals that persist throughout their lifespan. By differentiating into neurons, NSCs not only replenish lost neurons but also enhance the local microenvironment, promote angiogenesis, regulate inflammatory responses, and play an essential role in the repair of SCI (Zhao et al., 2021). Exogenous cell transplantation shows promise in SCI recovery. However, it faces challenges such as cancer risk, invasiveness, potential complications, and difficulties in controlling transplanted cell fate and long-term effects (Panayiotou and Malas, 2013; Liu and Chen, 2019).

In contrast, eNSCs in the spinal cord offer a safer and less invasive alternative for SCI treatment (Havelikova et al., 2022). With specific interventions, eNSCs can differentiate into neurons, reconstruct neural networks, and generate oligodendrocytes, thereby promoting the repair of SCI (Gilbert et al., 2022). Therefore, understanding the activation and differentiation mechanisms of eNSCs holds excellent potential for developing safer and more controllable therapeutic strategies, avoiding the risks associated with exogenous cell transplantation, and opening new prospects for neural regeneration.

### 3.2 eNSCs response after SCI

SCI activates the microenvironment of eNSCs, prompting these cells to proliferate rapidly in response to the injury (Li et al., 2024a). In the absence of injury, eNSCs are generally quiescent or proliferate very slowly (Horky et al., 2006; Ke et al., 2006). After SCI, the inflammatory response releases signaling molecules and growth factors that activate and promote the proliferation of eNSCs. Chemokines and components of the extracellular matrix guide eNSCs from their niches to the lesion site, where they differentiate into immature neurons (neuron-specific class III β-tubulin, Tuj1+), and mature neurons (microtubule-associated protein, MAP+), re-establishing functional neural networks in the host tissue (Samanta et al., 2015; Li et al., 2023). However, the neurogenesis process of eNSCs following SCI is complex and often insufficient for spontaneous neuronal differentiation (Duan et al., 2016; Xu et al., 2019). Post-injury, eNSCs predominantly differentiate into GFAP^+^, with approximately 95% of newly formed GFAP^+^ cells originating from eNSCs (Li et al., 2023). Among these, 53% are derived from the proliferation of resident GFAP^+^ cells, and the remaining 47% are from the differentiation of eNSCs. In contrast, oligodendrocyte differentiation is limited to less than 5%, and neuronal differentiation is almost negligible (Barnabe-Heider et al., 2010; Li et al., 2019; Li et al., 2023). Nevertheless, activated endogenous neural stem cells (eNSCs) exhibit a certain degree of regenerative potential in response to SCI, as evidenced by their activation, proliferation, and migration. These activated eNSCs originate from the ependymal region and migrate along vascular structures. The extracellular matrix, accumulating in the lesion core and adjacent peripheral zones, displays a centripetal-to-peripheral distribution pattern (Finkel et al., 2021). Within the lesion core, eNSCs predominantly differentiate into astrocytes, contributing to glial scar formation. In regions containing residual axons, a small subset differentiates into oligodendrocytes (Meletis et al., 2008). Neuronal differentiation is observed only in minimal numbers and is typically restricted to areas distal from the inflammatory core (Meletis et al., 2008). In contrast, non-activated eNSCs remain localized around the central canal in a quiescent state, serving as a potential reservoir for regeneration (Figure 1).

Therefore, directing the differentiation of eNSCs, particularly toward the neuronal lineage, is regarded as a pivotal strategy for enhancing spinal cord regeneration. By optimizing the injury microenvironment or targeting critical signaling pathways to reduce astrocytic differentiation and improve neurogenesis, it may be possible to reconstruct neural circuits and provide a more effective therapeutic approach for functional recovery after SCI.

## 4 Key mechanisms of eNSCs differentiation

**Figure 2:**
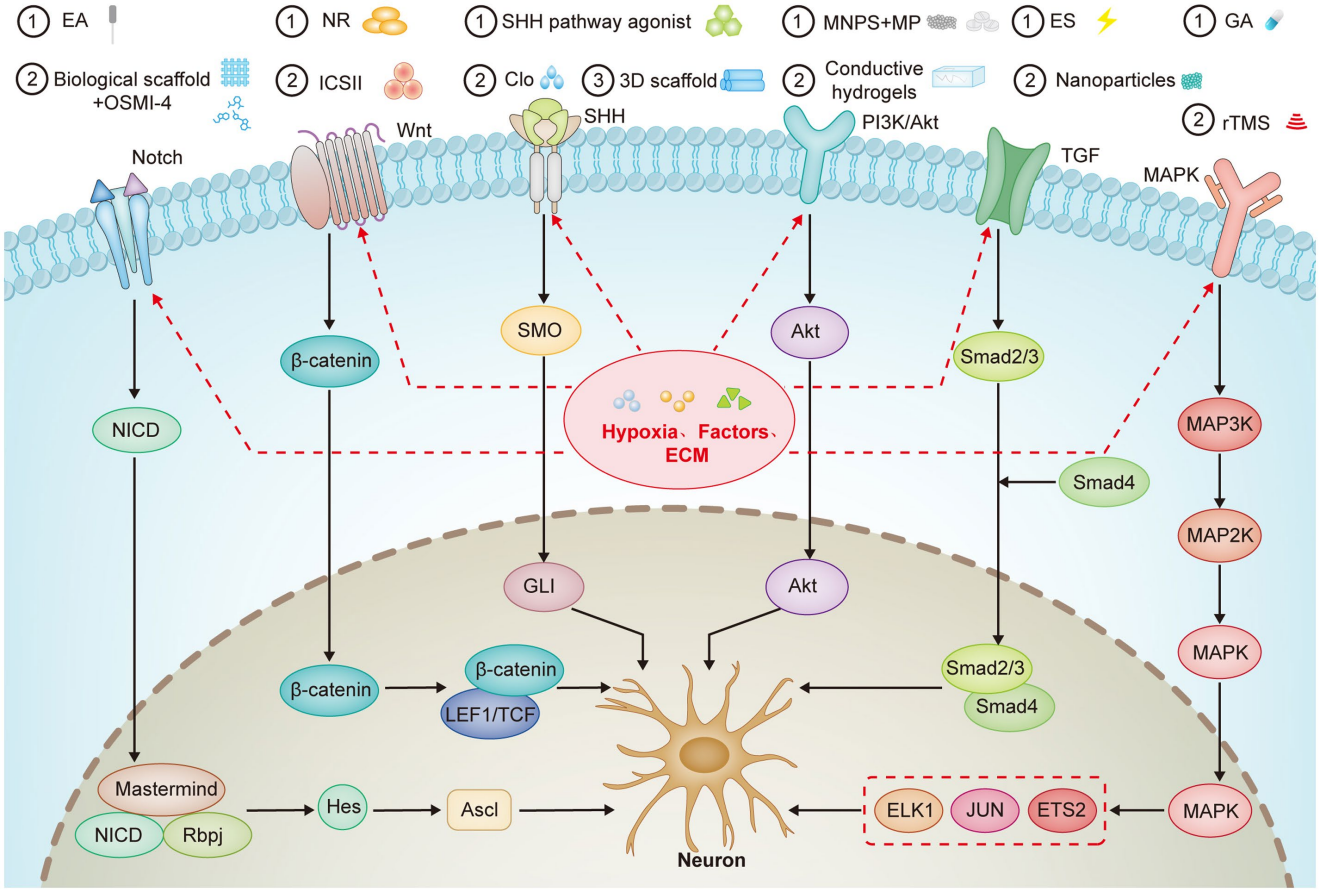
Key Signaling Pathways Involved in eNSCs Differentiation and Neural. This figure outlines the pivotal signaling pathways and their interactions in regulating the differentiation of eNSCs. The Notch, Wnt/β-catenin, SHH, PI3K/Akt, TGF-β, and MAPK pathways control eNSCs proliferation, differentiation, and fate determination. The paths are influenced by various microenvironmental factors, including hypoxia and ECM components, which modulate stem cell behavior and promote neural regeneration. Additionally, several therapeutic interventions such as EA, pharmacological agents, and biomaterials like conductive hydrogels are shown to interact with these pathways to enhance neurogenesis and SCI repair. The figure also highlights the interplay between hypoxia, ECM, and inflammatory factors in fine-tuning eNSCs differentiation towards neuronal or glial lineages, offering potential therapeutic strategies for neural regeneration in SCI and neurodegenerative diseases.

This section outlines the key signaling pathways in differentiating eNSCs, including Notch, Wnt/β-catenin, Sonic Hedgehog (SHH), PI3K/Akt, TGF-β, and MAPK. These pathways regulate the balance between NSC proliferation and their differentiation into neuronal or glial lineages. It also highlights the role of various microenvironmental factors, such as hypoxia, extracellular matrix components, inflammatory factors, and specific biochemical signals, as well as therapeutic strategies involving small molecules, nanoparticles, and scaffold-based approaches (Figure 2). These strategies are discussed for their potential to enhance neural repair and neurogenesis, particularly in SCI models.

### 4.1 Signaling pathways

#### 4.1.1 Notch signaling pathway

The Notch signaling pathway was discovered in *Drosophila melanogaster* over 80 years ago. It is critical in regulating numerous biological processes, including nervous system development. Studies showed that partial loss-of-function mutations in the Notch receptor gene result in defects in the wing margin of *Drosophila melanogaster* (Artavanis-Tsakonas et al., 1999). The Notch gene encodes a 300 kDa single-pass transmembrane receptor, which contains 36 epidermal growth factor (EGF)-like repeats and three cysteine-rich Notch/LIN-12 repeats (Artavanis-Tsakonas et al., 1999). This pathway is pivotal in developing the nervous system, particularly in regulating the proliferation, differentiation, and maintenance of the quiescent state of NSCs (Artavanis-Tsakonas et al., 1999; Nyfeler et al., 2005; Imayoshi et al., 2010; Basak et al., 2012). Activation of the Notch signaling pathway in NSCs occurs through the interaction between the Notch receptor and its ligand. This interaction leads to the cleavage of the receptor and the translocation of the Notch intracellular domain (NICD) into the nucleus, where it binds to the transcription factor recombination signal binding protein (Rbpj; Foley et al., 2024). Furthermore, the Mastermind protein is essential for stabilizing the NICD-Rbpj complex and enhancing the transcriptional activation of downstream target genes. This binding initiates the transcription of target genes, including the Hes, Her, and Hey families, which are essential for regulating the proliferation and quiescence of NSCs (Foley et al., 2024). Notch3 is primarily expressed in quiescent NSCs, while Notch1b expression is restricted to activated NSCs (Labusch et al., 2020). Disruption of Notch signaling, either through the removal of Notch3 or inhibition of Notch1, activates NSCs and promotes their differentiation (Chapouton et al., 2010; Marz et al., 2010; Alunni et al., 2013; Kawai et al., 2017; Engler et al., 2018). The Notch signaling pathway also regulates the expression of Hes1, which forms a negative feedback loop that further modulates NSCs proliferation and quiescence. The expression of Hes1 cyclically suppresses the expression of Ascl1, a key gene involved in neuronal differentiation. Inhibition of Notch signaling leads to reduced Hes1 expression, resulting in the sustained activation of Ascl1, which drives NSC differentiation into neurons (Imayoshi et al., 2013). This sustained Ascl1 expression is crucial for terminating the cell cycle and initiating neuronal differentiation, underscoring the central role of Ascl1 in regulating NSC fate (Imayoshi et al., 2013; Sueda et al., 2019).

Furthermore, the Notch signaling pathway regulates the expression of proteins such as Wnt4 and fucosyltransferase 9, which, by inhibiting Notch signaling, promote the differentiation of NSCs into neurons (Chen et al., 2023). This regulatory mechanism is critical in the context of SCI. For example, inhibiting Notch1 signaling has been shown to significantly enhance the differentiation of NSCs into neurons and glial cells, thereby accelerating the development of the nervous system (Chen et al., 2021). This is achieved by upregulating neuronal differentiation markers such as neurofilament (NF), GFAP, and galactosylceramidase (GALC). Researchers have developed a spinal cord-like bioprinted scaffold based on this mechanism, incorporating the small molecule drug OSMI-4 and supramolecular bioink (Liu et al., 2022). This approach effectively promotes the differentiation of NSCs into neurons by inhibiting Notch signaling, facilitating neural regeneration and motor function recovery in the complex microenvironment of SCI (Liu et al., 2022). This strategy holds significant promise as a therapeutic method for SCI repair.

Researchers have built on these findings to develop translational approaches that test Notch pathway modulation in clinical or experimental settings. Clinical studies have further explored the therapeutic potential of modulating Notch signaling in SCI. Electroacupuncture (EA), for example, has been shown to inhibit the activation of Notch signaling through regulation of the H19/EZH2 axis. This modulation promotes NSC proliferation and enhances their differentiation into neurons while suppressing cell apoptosis, ultimately promoting SCI repair and improving functional recovery (Geng et al., 2021).

In conclusion, the Notch signaling pathway plays a critical role in regulating the proliferation, differentiation, and maintenance of the quiescent state of NSCs. Inhibiting Notch signaling promotes the differentiation of NSCs into neurons and glial cells, effectively accelerates neurogenesis, and significantly enhances SCI repair. These findings suggest that targeting Notch signaling may be a valuable strategy for advancing therapeutic approaches to SCI.

#### 4.1.2 Wnt/β-catenin signaling pathway

The Wnt signaling pathway includes 19 mammalian genes encoding secreted glycoproteins (Wnt ligands) and their Frizzled (Fzd) receptors. It is classified into three branches: (1) the canonical Wnt/β-catenin pathway, (2) the planar cell polarity (Wnt/PCP) pathway, and (3) the Wnt/Ca^2+^ pathway (Xu et al., 2024). In the canonical Wnt/β-catenin signaling cascade, Wnt ligands bind to the Fzd receptors and low-density lipoprotein receptor-related proteins 5/6 (LRP5/6), leading to the activation of the intracellular Dishevelled (Dvl) proteins. This interaction prevents the degradation of β-catenin by the GSK-3β/APC/CK1α/Axin complex, thereby promoting its accumulation in the cytoplasm (Bem et al., 2019; Kriska et al., 2021). Subsequently, β-catenin is transported into the nucleus, where it interacts with transcription factors LEF1/TCF to initiate the transcription of Wnt target genes (Xu et al., 2024). The Wnt/Ca^2+^ pathway is activated when Wnt ligands bind to the Fzd receptors, activating phospholipase C (PLC). This activation releases calcium ions from the endoplasmic reticulum, which modulates intracellular calcium concentrations (Kohn and Moon, 2005). The Wnt/PCP pathway regulates cell polarity and migration by activating small GTPases, such as RhoA and Rac, following the binding of Wnt ligands to Fzd receptors. This activation subsequently triggers Jun N-terminal kinase (JNK), regulating cellular motility (Chiareli et al., 2022).

In the nervous system, the Wnt/β-catenin signaling pathway is crucial for the proliferation and differentiation of NSCs. It plays a central role in maintaining the balance between progenitor cell proliferation and neural differentiation during the development of NSCs, cortical cells, and dopaminergic precursor cells, thus supporting the development and maintenance of functional neural circuits (L'Episcopo et al., 2014; Noelanders and Vleminckx, 2017; Mishra et al., 2019). The activation of the Wnt/β-catenin pathway is essential for maintaining adult NSCs and contributes to stem cell homeostasis across various tissues (Bowman et al., 2013). Studies have shown that knockout of the Ctnnb1 gene, a key component in the Wnt pathway, leads to either expansion of the progenitor cell pool or reduced proliferation, underscoring the critical role of β-catenin in regulating NSC proliferation and differentiation (Bengoa-Vergniory and Kypta, 2015).

In the context of SCI repair, activation of the Wnt/β-catenin signaling pathway has been shown to promote the proliferation and differentiation of NSCs. For example, nicotinamide riboside (NR) activates the Wnt signaling pathway by targeting the LGR5 gene, thereby enhancing the proliferation and differentiation of eNSCs and improving neural repair after SCI (Zhang et al., 2024b). Furthermore, Icarisid II (ICS II) has been found to promote the activation of the Wnt/β-catenin pathway by upregulating Wnt-3a expression, phosphorylating GSK-3β, and enhancing the nuclear translocation of β-catenin (Xiao et al., 2021). These events facilitate the proliferation of NSCs and neuronal differentiation. Conversely, miR-103-3p inhibits the Wnt/β-catenin pathway by targeting the Ndel1 protein, leading to suppressed proliferation, enhanced differentiation, and increased apoptosis of NSCs (Li et al., 2022). This highlights the critical role of this pathway in regulating NSC fate.

In summary, the Wnt/β-catenin signaling pathway is integral to the proliferation and differentiation of NSCs. Modulating this pathway makes it possible to promote NSC proliferation and neuronal differentiation and identify novel therapeutic strategies and potential targets for neural repair and regeneration following SCI.

#### 4.1.3 SHH signaling pathway

The Sonic Hedgehog (SHH) signaling pathway is pivotal in neural development, particularly in regulating neurogenesis within the adult subventricular zone (SVZ). SHH governs NSC behavior by modulating their proliferation and differentiation into neuronal lineages (Wang et al., 2022). This pathway is classified into canonical and noncanonical types. The canonical path involves the 12-transmembrane receptor Patched1 (PTCH1), the 7-transmembrane receptor Smoothened (SMO), and GLI family transcription factors (GLI1, GLI2, and GLI3; Espinosa-Bustos et al., 2019; Yang et al., 2021a). Activation of SHH signaling occurs through SMO, which promotes the proliferation and clonal expansion of NSCs and drives their differentiation into neuronal lineages (Vicario et al., 2019). The nuclear translocation of GLI1 mainly mediates this process. The noncanonical SHH signaling pathway includes SMO-dependent but GLI-independent pathways, PTCH-mediated pathways, and SMO-independent GLI activation (Rimkus et al., 2016). SHH signaling enhances Ca^2+^ activity in NSC primary cilia by promoting TRPC3 channel influx and intracellular Ca^2+^ release. This process downregulates Sox2 and simultaneously upregulates neurogenic genes, thereby driving neuronal differentiation (Shim et al., 2023). Furthermore, recent studies have shown that tissue transglutaminase (TG2)-modified ectodermal mesenchymal stem cells (TG2-EMSCs) secrete elevated levels of SHH protein. This sustained SHH release further amplifies NSC differentiation into neuronal lineages (Shi et al., 2021). These findings highlight the SHH pathway’s crucial role in NSC differentiation, emphasizing its therapeutic potential in neural development and injury repair.

In addition to its role in neurogenesis, SHH signaling has therapeutic potential. In stroke models, SHH pathway agonists improve motor and cognitive recovery by enhancing NSC survival and differentiation in the SVZ and subgranular zone, significantly increasing neuroblast and neuron generation (Jin et al., 2017). The transcription factor YAP regulates SHH effector Gli2 through TRIP6-mediated LATS1/2 kinase activation, maintaining NSC quiescence while suppressing differentiation (Li et al., 2024b). Clobetasol propionate (Clo) activates SHH signaling, promoting NSC differentiation into neurons and oligodendrocytes while inhibiting the formation of astrocytes (Shi et al., 2019). Recent advances include the development of polylactic acid (PLA) 3D scaffolds functionalized with gold nanoparticles, nerve growth factor peptides, and SHH coatings. These scaffolds significantly enhance human NSC proliferation and differentiation, with SHH-coated scaffolds upregulating motor neuron-specific markers (HB9 and TUJ-1), demonstrating their potential for spinal cord injury (SCI) repair (Kim et al., 2022).

In conclusion, SHH signaling is pivotal in NSC proliferation, maintenance, and neuronal differentiation. This highlights its significant therapeutic potential for stroke, neurodevelopmental disorders, and SCI. Future studies should optimize SHH modulation strategies, such as controlled delivery systems or combinatorial therapies, to enhance functional recovery in SCI models.

#### 4.1.4 PI3K/Akt signaling pathway

The PI3K/Akt pathway is a critical intracellular signaling cascade regulating cell survival, proliferation, metabolism, and angiogenesis. Within the nervous system, this pathway exerts pivotal control over neuronal survival, neurogenesis, and apoptotic processes (Katso et al., 2001; Engelman et al., 2006; Yang et al., 2019). The path consists of two main components: PI3K and Akt. PI3K, a lipid kinase family member, comprises regulatory subunits p55 and p85 and a catalytic subunit p110, with class I PI3K being the most central (Cantley, 2002; Guo et al., 2024). Upon activation by upstream signals, such as GPCRs or RTKs, PI3K catalyzes the conversion of phosphatidylinositol-4,5-bisphosphate (PIP2) to phosphatidylinositol-3,4,5-trisphosphate (PIP3), which recruits Akt (Tian et al., 2023). Akt, a serine/threonine kinase, regulates key cellular functions and consists of a Pleckstrin homology (PH), central, and regulatory carboxyl-terminal domain (Hanada et al., 2004). AKT activity is triggered by the accumulation of PIP3, which activates the catalytic domain of AKT via phosphoinositide-dependent kinase 1 (PDK1) at the Thr308 site, a critical step for AKT activation (Guo et al., 2024). Additionally, PIP3 activates AKT at the Ser473 site through mTORC2, further enhancing its activity (Miricescu et al., 2020). These post-translational modifications enable Akt to orchestrate diverse cellular responses through downstream effector regulation.

Emerging evidence highlights the pathway’s crucial role in neural stem cell (NSC) dynamics and CNS repair. Experimental models demonstrate that PI3K/Akt activation enhances NSC proliferation while biasing differentiation toward neuronal lineages over glial fates. For instance, magnetic nanoparticle (MNPs)-mediated delivery of methylprednisolone (MP) combined with magneto-mechanical stimulation activates this pathway, achieving significant axonal regeneration and functional recovery in SCI models (Zhang et al., 2024d). Similarly, injectable conductive hydrogels promote endogenous NSC-mediated neurogenesis and remyelination through PI3K/Akt activation, facilitating structural and functional neural repair post-SCI (Luo et al., 2022). Pharmacological interventions further validate this pathway’s therapeutic potential. Magnesium lithospermate B (MLB) stimulates pluripotent NSC proliferation and neuronal differentiation *in vitro* via PI3K/Akt signaling, with *in vivo* administration enhancing hippocampal neurogenesis (evidenced by increased Ki67+/Thy1 + cells) and improving motor function in injury models (Zhang et al., 2018). In addition, in vivo experimental results have shown that the delivery system combining MNPs with MP does not induce significant inflammatory responses or organ toxicity, nor has tumor-like hyperplasia been observed (Zhang et al., 2024d). This is likely attributed to its precise local stimulation and controlled dosing mechanisms. Following the application of an injectable conductive hydrogel, a significant increase in the proportion of newly generated neurons within the injured area was observed, which was associated with the synergistic activation of the PI3K/Akt signaling pathway. Moreover, the Basso, Beattie, and Bresnahan (BBB) locomotor scores were markedly improved, and this functional enhancement was sustained over time (Luo et al., 2022).

Meanwhile, while enhancing neurogenesis in the hippocampus, MLB did not induce significant liver or kidney function abnormalities, suggesting favorable preliminary biosafety (Zhang et al., 2018). However, its long-term toxicity remains to be further investigated. These findings further demonstrate the critical role of the PI3K/Akt signaling pathway in promoting NSCs differentiation and neuroregeneration, particularly highlighting its significant potential in SCI repair.

In summary, the PI3K/Akt signaling pathway is central in regulating the differentiation of eNSCs, neuroregeneration, and SCI repair. By activating this pathway through multifunctional delivery systems, conductive hydrogels, or small-molecule drugs, it is possible to significantly enhance the differentiation of NSCs into functional neurons and promote axonal regeneration, remyelination, and neural network remodeling. These strategies have demonstrated promising efficacy and application prospects in both in vitro and in vivo studies, offering safe and effective potential therapeutic approaches for SCI repair and laying a solid foundation for their clinical translation.

#### 4.1.5 TGF-β signaling pathway

The Transforming Growth Factor-β (TGF-β) superfamily includes more than 40 ligands, such as TGF-βs, Growth Differentiation Factors (GDFs), Nodal-related proteins, Bone Morphogenetic Proteins (BMPs), and Activins. These ligands regulate biological processes, including gene expression, cell proliferation, differentiation, and development (Feng and Derynck, 2005). They govern key functions such as cell growth, survival, migration, fate determination, and lineage differentiation during embryonic development and adult tissue homeostasis (Morikawa et al., 2016; Tamayo et al., 2018). TGF-β signaling is initiated when TGF-β ligands bind to a heterotetrameric receptor complex on the cell surface, consisting of type I (TβRI) and type II (TβRII) serine/threonine kinase receptors. Ligand binding to TβRII activates TβRI, which triggers either Smad-dependent or Smad-independent pathways (Saranya and Selvamurugan, 2024). In the Smad-dependent pathway, the activated TβRI phosphorylates receptor-regulated Smads (R-Smads), such as Smad2/3 (for TGF-β) or Smad1/5/8 (for BMP signaling). These R-Smads then form complexes with the common Smad (Co-Smad, such as Smad4) and translocate to the nucleus to regulate the transcription of target genes (Nickel and Mueller, 2019; Sanchez-Duffhues et al., 2020; Tzavlaki and Moustakas, 2020). Inhibitory Smads (I-Smads, such as Smad6/7) negatively regulate the TGF-β pathway through feedback inhibition (Murayama et al., 2020; Park et al., 2021). Additionally, noncanonical TGF-β/BMP pathways involve various signaling branches such as TAK1, MAPK, ERK1/2, JNK, p38 MAPK, PI3K/Akt, RhoA, and PP2A, which can independently mediate signal transduction or synergize with the Smad pathway (Wu et al., 2019b; Song et al., 2020; Tzavlaki and Moustakas, 2020; Yue et al., 2020; Zheng et al., 2022). TGF-β superfamily ligands regulate cellular biological functions through Smad-dependent and Smad-independent pathways, extensively participating in cell differentiation and other physiological processes. The activation of these signaling pathways relies on the formation and phosphorylation of ligand-receptor complexes. It involves downstream effectors such as R-Smads, which cooperate with nuclear transcription factors and cofactors to modulate gene expression.

In differentiating NSCs, the TGF-β signaling pathway plays a critical role. Studies have shown that TGF-β synergizes with signals such as Wnt-3a and Ephrin-B2 to promote neuronal differentiation and maturation while coordinating NSC fate determination alongside Bone Morphogenetic Protein 4 (BMP4; Muckom et al., 2019). For instance, an electrically stimulated Zn^2+^-controlled release strategy using Zn-PDA@BT nanoparticles activates both the TGF-β and p53 pathways, significantly promoting the differentiation of embryonic eNSCs into neurons while inhibiting astrocyte differentiation, thereby replenishing lost neurons and enhancing functional recovery in spinal cord injury (SCI) models (Han et al., 2024). This approach provides a novel potential strategy for neural regeneration in SCI repair.

In summary, the TGF-β signaling pathway regulates eNSCs differentiation through Smad-dependent and Smad-independent mechanisms. It promotes neuronal generation while inhibiting astrocyte formation. Combining this approach with Zn^2+^-controlled release technology accelerates neural regeneration and functional recovery in SCI repair. Modulating the TGF-β pathway offers novel therapeutic strategies for SCI repair and treating neurodegenerative diseases, demonstrating broad potential for clinical applications.

#### 4.1.6 MAPK signaling pathway

Mitogen-activated protein Kinases (MAPKs) are a family of highly conserved serine–threonine kinases and serve as one of the key signaling pathways in eukaryotic cells. MAPKs integrate extracellular signals with core cellular processes by responding to various stimuli, coordinating cellular responses, and regulating cell proliferation, differentiation, growth, apoptosis, and stress responses (Lavoie et al., 2020; Ronkina and Gaestel, 2022; Lin et al., 2025). As such, the MAPK pathway is crucial for determining cell fate.

The MAPK pathway is activated through a phosphorylation cascade involving three key kinases: MAP3K, MAP2K, and MAPK. MAP3K, the most upstream kinase, senses intracellular and extracellular signals and activates MAP2K through phosphorylation (Lin et al., 2025). MAP2K then activates MAPK by phosphorylating conserved tyrosine and threonine residues within MAPK’s activation loop (Cappello et al., 2016). This allows MAPK to regulate multiple substrates and determine cellular fate in response to signals.

The MAPK signaling pathway in mammals is divided into four major subfamilies: JNK, p38, BMK1 (ERK5), and ERK (Farooq et al., 2001). The ERK family consists of ERK1 and ERK2, the first members identified in the classical Ras–Raf-MAPK signaling pathway. Upon activation by various growth factors, cytokines, mitogens, and hormones, ERK1/2 participates in signal transduction by phosphorylating intracellular kinases, thus playing a critical role in cell differentiation, growth, and proliferation (Dimri and Satyanarayana, 2020; Lavoie et al., 2020). The JNK family, comprising JNK1, JNK2, and JNK3, is classified as stress-activated protein kinases (SAPKs). These kinases primarily respond to inflammatory cytokines, environmental stress, GPCR agonists, and growth factors, regulating transcription factors (e.g., ELK1, JUN, ETS2) and downstream kinases to modulate physiological processes such as survival, proliferation, differentiation, and apoptosis (Lavoie and Therrien, 2015; Wu et al., 2019a; Hammouda et al., 2020). The p38 family includes four subtypes: p38α, p38β, p38γ, and p38δ. Their activation is triggered by stressors such as UV radiation, osmotic shock, hypoxia, inflammation, and phosphorylation by ERK3/6 (Lin et al., 2025). ERK5, or BMK1, regulates transcriptional processes by binding to DNA through its non-catalytic region (Kasler et al., 2000).

In neurobiology, the MAPK pathway plays a key role in the differentiation and proliferation of NSCs. For example, gallic acid (GA) from tea leaves promotes the differentiation of NSCs into immature neurons. It enhances their proliferative capacity by activating the MAPK/ERK pathway, underlining the importance of this pathway in regulating NSC differentiation and suggesting a potential molecular target for neural repair (Jiang et al., 2021b). Wnt5a promotes NSC differentiation into neurons by upregulating miRNA200b-3p expression via the MAPK/JNK pathway and inhibiting RhoA/Rock signaling (Li et al., 2020a; Li et al., 2020b). Additionally, Wnt4 enhances NSC differentiation efficiency by activating both the MAPK/JNK and β-catenin pathways while suppressing Notch signaling, particularly the nuclear binding of NICD. This approach significantly improves spinal cord repair and motor function recovery in SCI models (Li et al., 2020a; Li et al., 2020b). These studies further emphasize the central role of the MAPK pathway in NSCs differentiation and the repair of SCI by eNSCs. Furthermore, repetitive transcranial magnetic stimulation (rTMS) promotes NSC proliferation and neuronal differentiation while inhibiting glial differentiation by activating the MAPK pathway in a Ca^2+^-dependent manner. This accelerates neurological recovery after intracerebral hemorrhage (ICH), emphasizing the MAPK pathway’s critical role in neural repair (Cui et al., 2019).

Additionally, repetitive transcranial magnetic stimulation (rTMS) significantly enhances NSC proliferation and neuronal differentiation while inhibiting glial differentiation by activating the MAPK signaling pathway in a Ca^2+^-dependent manner. This accelerates neurological recovery after intracerebral hemorrhage (ICH), underscoring the critical role of the MAPK pathway in neural repair.

### 4.2 Other regulatory factors and microenvironment

The proliferation and differentiation of NSCs are governed by various microenvironmental cues, including hypoxia, extracellular matrix (ECM) components, inflammatory cytokine gradients, and specific biochemical factors. These elements modulate NSC dynamics through distinct molecular mechanisms, critically influencing neurogenic potential during neural regeneration. Current research prioritizes decoding these regulatory networks to develop targeted interventions for neural repair.

#### 4.2.1 Hypoxia and NSCs regulation

Hypoxia, as a common microenvironmental factor, significantly influences the proliferation and differentiation of NSCs. Hypoxia significantly influences the proliferation and differentiation of NSCs by activating key molecular pathways such as Notch, Wnt/β-catenin, and PI3K/Akt, as well as regulating cell cycle proteins, erythropoietin, and microRNA expression (Fan et al., 2023). Its effects depend on oxygen concentration, duration of hypoxia, tissue origin of NSCs, and their hypoxia tolerance thresholds, providing important insights for the clinical application and therapeutic potential of eNSCs (Fan et al., 2023). Additionally, hypoxia-inducible factor HIF-1α, through adenovirus-mediated gene expression, significantly promotes the proliferation, migration, and neuronal differentiation of eNSCs while increasing the levels of DCX and BrdU/DCX-positive cells, thereby improving neurological deficits. This further underscores the critical role of HIF-1α in neural repair (Yu et al., 2013).

#### 4.2.2 ECM and NSCs regulation

The role of the ECM in the proliferation and differentiation of NSCs cannot be overlooked. The ECM influences the behavior and differentiation of NSCs through its specific structural and biochemical properties, including mesh-like, fibrous, and tubular forms, regulating neurogenesis and regeneration in the central and peripheral nervous systems. At the same time, the anisotropic design of engineered biomimetic topological structures further enhances their effectiveness in promoting neuronal differentiation (Jain et al., 2020). Further studies have revealed that natural extracellular matrix-based conductive hydrogels can promote the differentiation of eNSCs into neurons, inhibit glial cell differentiation, and activate the PI3K/AKT and MEK/ERK pathways, thereby achieving myelinated axonal regeneration and motor function recovery. This provides an ideal biomaterial for treating traumatic SCI (Luo et al., 2022). Notably, ECM proteins inherently present in the injury site, such as laminin, fibronectin, and chondroitin sulfate proteoglycans (CSPGs), play critical roles during the remodeling process following neural injury. These proteins constitute damage-associated molecular patterns (DAMPs), activate local inflammatory responses, and influence the behavior of eNSCs (Siebert et al., 2014). CSPGs are widely recognized for their inhibitory effects on axonal regeneration and their ability to restrict the differentiation of NSCs into neurons. In contrast, laminin facilitates neuronal differentiation of eNSCs by promoting cell adhesion and inducing the expression of neuron-specific genes (Barros et al., 2020). Therefore, dynamic regulation of ECM components within the injury microenvironment may be key to optimizing strategies for neural repair. Studies have shown that implantation of an aligned collagen scaffold loaded with cetuximab, an inhibitor of the epidermal growth factor receptor (EGFR), not only promotes neuronal differentiation of NSCs but also reduces CSPG deposition, improves the local microenvironment, and significantly enhances neural regeneration and motor function recovery (Li et al., 2017). These findings suggest that functional scaffolds combined with bioactive regulatory factors can effectively activate the regenerative potential of eNSCs and support spinal cord injury repair.

#### 4.2.3 Inflammatory factors and NSC regulation

Inflammatory factors such as IL-1β, TNF-α, and IFN-γ alter the NSCs microenvironment by activating pro-inflammatory signaling pathways, inhibiting their proliferation, migration, and differentiation, thereby limiting their neuro regenerative potential. These factors may contribute to the decline of NSCs, further impacting the efficiency of neural repair processes. Following injury, activated microglia and infiltrating macrophages become significant sources of pro-inflammatory cytokines. Among these, tumor necrosis factor-alpha (TNF-α) and interleukin-6 have been shown to significantly inhibit the neuronal differentiation of NSCs by activating the NF-κB signaling pathway (Carpentier and Palmer, 2009). Studies further indicate that TNF-α secreted by microglia markedly reduces the survival rate of dopaminergic precursor cells and impairs their efficiency in differentiating into neurons, highlighting the critical inhibitory role of TNF-α in regulating NSCs fate (Wenker et al., 2023). SF/BP/GA hydrogel inhibits inflammatory responses to optimize the microenvironment at the injury site. It induces macrophage polarization toward the anti-inflammatory M2 phenotype, promoting the differentiation of eNSCs and significantly improving neural signal transmission and tissue regeneration, thereby effectively accelerating repair (Zhang et al., 2024a).

#### 4.2.4 Specific biochemical factors and NSC differentiation

In addition to microenvironmental factors, specific biochemical factors play a crucial role in differentiating NSCs by regulating signaling pathways and gene expression. For example, neurotrophin-3 (NT-3) significantly enhances the viability and differentiation capacity of NSCs, with sustained release through chitosan carriers effectively increasing the proportion of NSCs differentiating into neurons, including GABAergic and cholinergic neurons (Li et al., 2009). Additionally, insulin-like growth factor-1 (IGF-1), when immobilized on PLGA/GO electrospun nanofibers, significantly promotes the survival, proliferation, and differentiation of NSCs, demonstrating great potential for neuroprotection and neurogenic effects in neural implants (Qi et al., 2019). Interferon-γ (IFN-γ) significantly promotes the differentiation of adult NSCs into neurons, enhances the generation of βIII-tubulin-positive neurons, and facilitates neurite outgrowth and an increase in neurite number (Wong et al., 2004).

In summary, hypoxia, ECM, inflammatory microenvironments, and specific biochemical factors collectively regulate the proliferation, differentiation, and neuroregeneration of eNSCs through various mechanisms. Modifying these microenvironmental factors provides a deeper understanding of NSCs research and offers essential strategies and potential therapeutic directions for SCI repair.

## 5 Future translational applications and challenges

**Figure 3:**
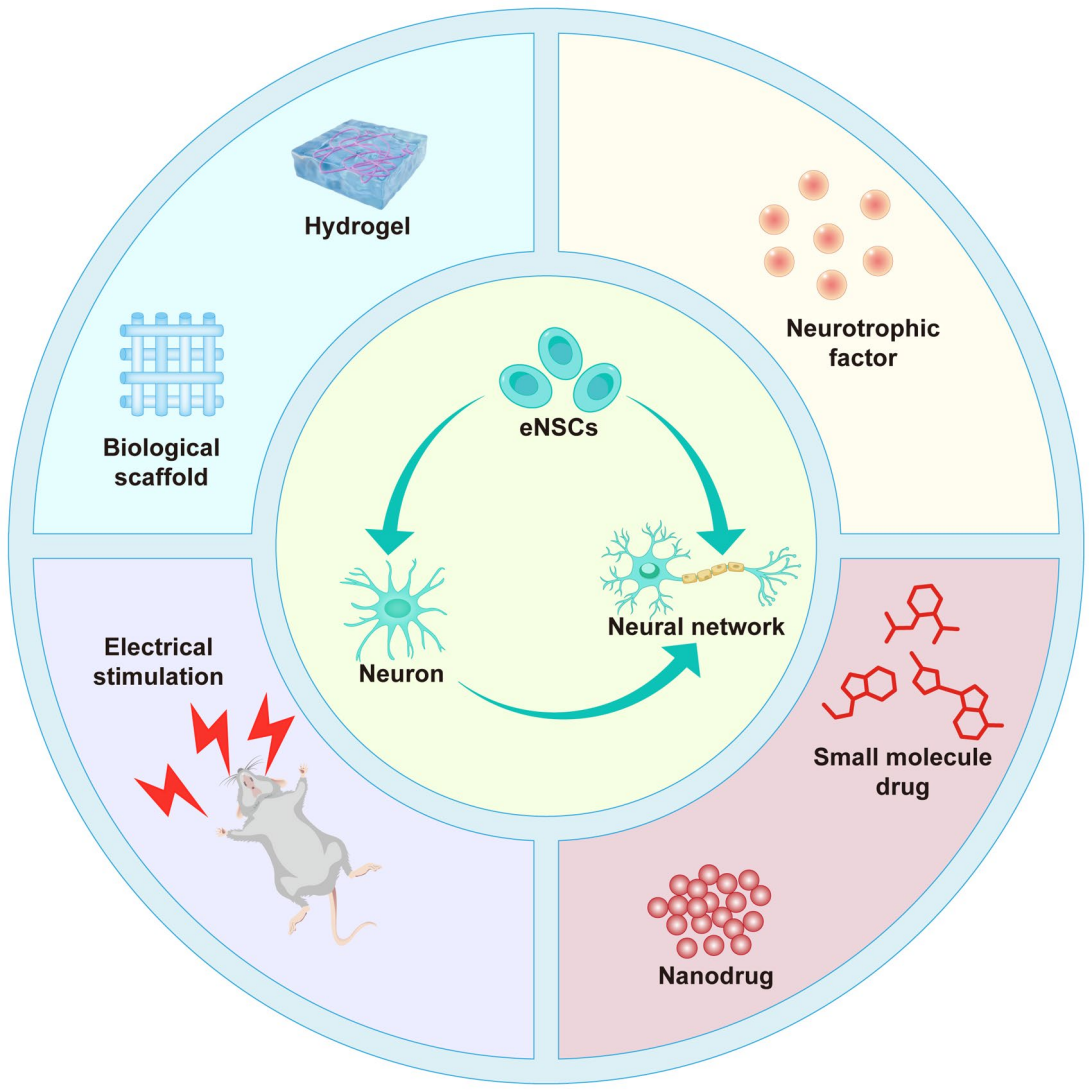
Schematic illustration of multimodal therapeutic strategies for SCI repair. This figure highlights various approaches, including the use of biological scaffolds (e.g., hydrogels and collagen-based structures), neurotrophic factors (e.g., NT-3), electrical stimulation, and nanodrug systems to promote the differentiation and migration of eNSCs into functional neurons and enhance the formation of neural networks. These strategies collectively aim to overcome the limitations of single therapies by providing structural support, delivering pharmacological agents, and promoting regeneration and functional recovery following SCI.

Despite significant progress in the field of SCI repair, multiple challenges remain. The inherent limitations in the differentiation capacity of eNSCs, coupled with factors such as excessive immune responses, inhibitory factors, and glial scarring in the traumatic environment, restrict the effectiveness of single therapeutic strategies (Li et al., 2023). Therefore, overcoming these obstacles and achieving more effective repair has become a critical issue in SCI research. In recent years, advancements in biomaterials have provided new hope for combined therapies in SCI, particularly multimodal approaches that integrate pharmacological regulation, physical stimulation, and scaffold implantation (Figure 3). Studies in animal models have shown that these therapeutic strategies exhibit significant repair effects. For example, scaffold implantation provides structural support and significantly enhances local treatment efficiency through controlled drug release (Li et al., 2019; Zhang et al., 2019). Among these, research on aligned collagen-fibrin (Col-FB) hydrogels has demonstrated that their combination of stretchability, adhesiveness, and spatiotemporal delivery of SDF1α/paclitaxel (PTX) effectively promotes the migration and differentiation of eNSCs, significantly improving motor function (Chen et al., 2022). In further studies, collagen scaffolds modified with cetuximab and paclitaxel have shown promising results in rat SCI models, significantly reducing glial scar formation and promoting axonal regeneration (Fan et al., 2018b). Additionally, loading small-molecule drugs (e.g., LDN193189, CHIR99021) into biomaterials can further activate differentiation signaling pathways in NSCs, inhibit astrocyte generation, and promote the differentiation of NSCs into neurons (Yang et al., 2021b). In recent years, nano-drug delivery systems have also demonstrated significant potential. For example, polysialic acid (PSA)-loaded minocycline nanoparticles have shown excellent anti-inflammatory effects and neuroprotective properties in SCI models while promoting the migration of NSCs and the regeneration of neurons (Wang et al., 2019). NT-3 has recently garnered widespread attention due to its ability to promote oligodendrocyte proliferation and neuronal survival without causing side effects such as pain or spasms (Cong et al., 2020). A study developed a biphasic silk fibroin hydrogel scaffold (DPSH) encapsulating NT-3 and angiotensin (Ang-1-7) microspheres to promote SCI repair. The results showed that NT-3 significantly enhanced the differentiation capacity of NSCs into neurons, improved neuronal survival and regeneration, and enhanced motor function (Zhang et al., 2024c). Electrical stimulation (ES) combined with conductive hydrogels capable of *in situ* power generation significantly promoted the differentiation of eNSCs and accelerated SCI repair. This hydrogel can provide adjustable ES to the injured area under non-invasive conditions, generating alternating current through capacitive coupling, promoting remyelination and axonal repair, and demonstrating significant repair potential (Wu et al., 2024). Although these multimodal therapies have shown significant repair potential in animal models, they still face numerous challenges in clinical applications. Currently, the optimal source of NSCs in the adult spinal cord and their dynamic regulatory mechanisms remain unclear, and this represents one of the major bottlenecks limiting the efficiency of clinical translation (Garcia-Alias et al., 2009; Fouad et al., 2013; Li et al., 2023). Although current studies generally regard the central canal region of the spinal cord as a major reservoir of NSCs, many questions remain regarding their activation mechanisms, migration dynamics, and differentiation potential under conditions of injury or disease, necessitating further investigation (Yang et al., 2021b). In addition, emerging technologies such as omics approaches, single-cell sequencing, and lineage tracing have identified novel therapeutic targets. Elucidating the underlying mechanisms may enable precise regulation of eNSCs differentiation, facilitating the generation of multiple cell types such as neurons, astrocytes, and oligodendrocytes in appropriate proportions to promote effective spinal cord reconstruction (Cavone et al., 2021; Stenudd et al., 2022). On the other hand, improving the scientific rigor and clinical applicability of SCI research is equally critical. Given the high heterogeneity and individual variability associated with SCI, it is recommended to narrow the inclusion time window, incorporate objective biomarkers for injury assessment, and establish more accurate prognostic evaluation systems. These measures may help select well-characterized patient subgroups with controlled pathology, thereby enhancing study homogeneity and the comparability of therapeutic interventions (Hu et al., 2023). Moreover, although multimodal therapies have shown promising outcomes in animal models, their clinical translation remains challenging due to highly controlled experimental conditions and interspecies differences (Stewart et al., 2023). Therefore, Future research should focus on developing large animal models, humanized systems, organoids, and *in vitro* three-dimensional culture platforms that better replicate human pathological conditions to improve the clinical relevance of preclinical findings. Meanwhile, optimizing treatment timing, dosage, and delivery methods will be an important direction for future research. By precisely regulating the activation of signaling pathways, the spatiotemporal delivery of drugs, and the personalized treatment plans, we can further enhance the differentiation efficiency and functional recovery of neural stem cells, thereby providing broader application prospects for SCI repair (Table 1). In summary, although multimodal therapeutic strategies have demonstrated promising regenerative potential in animal models, their clinical translation remains hindered by multiple challenges, including unclear mechanisms, model limitations, and significant individual variability. Future efforts should focus on elucidating the regulatory mechanisms of NSCs, integrating high-throughput technologies, optimizing the properties of biomaterials, and incorporating precise clinical evaluation systems. These approaches are essential to achieve accurate control of NSCs fate and functional reconstruction, ultimately advancing the clinical application and translational implementation of SCI therapies.

**Table 1:** Translational Applications and Challenges in Future Therapies for SCI.

Type of Study	Phase	Key Findings	Challenges	References
Collagen hydrogel co-loaded with SDF1α and PTX	Animal model	Promotes migration and differentiation of eNSCs, improving motor function.	The response of human-derived NSCs remains unpredictable, and the optimal timing for therapeutic intervention requires further refinement.	Chen et al. (2022)
Cetuximab/paclitaxel-modified collagen scaffold	Animal model	Reducing glial scar formation and promoting axonal regeneration.	The mechanisms underlying glial responses are complex, and their clinical safety and efficacy remain uncertain.	Fan et al. (2018b)
Small-molecule compound	Animal model	Activate NSCs differentiation signaling while inhibiting astrocyte generation.	Achieving precise control over drug dosage and spatiotemporal delivery remains technically demanding and limits therapeutic efficacy.	Yang et al. (2021b)
PSA-minocycline nano-drug	Animal model	Demonstrates significant anti-inflammatory effects and promotes neuroprotection and regeneration.	The biocompatibility and long-term stability of nanocarriers have yet to be fully verified.	Wang et al. (2019)
NT-3/Ang-1-7 biphasic silk fibroin hydrogel scaffold	Animal model	Promotes neuronal differentiation and survival and enhances motor function.	Variability in the degradability of biomaterials and individual adaptability remains a concern.	Zhang et al. (2024c)
ES Combined with Conductive Hydrogels	Animal model	Accelerate NSCs differentiation and promote remyelination.	The lack of standardized electrical stimulation parameters poses significant challenges for clinical control.	Wu et al. (2024)
Multi-omics integrated with single-cell sequencing for mechanistic investigation of therapeutic interventions	Basic research	Elucidating the regulatory network governing the fate determination of NSCs may enhance the precision of therapeutic interventions.	The interpretation of results remains complex, and the translational pathway requires further clarification.	Cavone et al. (2021); Stenudd et al. (2022)
Arc-EX non-invasive electrical stimulation device	Phase II clinical trial	Significant improvements in limb function were observed, with 72% of patients demonstrating enhanced arm strength and functionality within 2 months.	Long-term efficacy and applicability across different types of spinal cord injuries remain to be validated.	Moritz et al. (2024)
Local delivery of VX-210 (a Rho signaling pathway inhibitor)	Phase II clinical trial	Promotes axonal regeneration and improves motor function.	Clinical efficacy remains limited, highlighting the need to optimize delivery methods and dosing strategies.	Fehlings et al. (2021)

## 6 Summary and future perspectives

This article systematically reviews the critical role of endogenous eNSCs in SCI repair, exploring their activation, proliferation, and differentiation mechanisms, particularly the key regulatory pathways driving neuronal differentiation. Through an in-depth analysis of multiple signaling pathways, such as Notch, Wnt/β-catenin, and Sonic Hedgehog, the potential of eNSCs in post-SCI repair has been revealed. Additionally, micro-environmental factors such as hypoxia, the extracellular matrix, and inflammatory responses play crucial roles in regulating the proliferation and differentiation of eNSCs.

Despite significant advances in basic research, the efficiency of the directed differentiation of eNSCs into functional neurons remains low. Moreover, the underlying biological regulatory mechanisms are not fully understood, and numerous challenges hinder their clinical translation. Factors such as glial scar formation, the persistence of an inflammatory microenvironment, and the lack of efficient therapeutic delivery platforms severely limit the recovery of neurological function.

Future research should prioritize the development of multimodal combinatorial intervention strategies, including drug targeting, physical stimulation (such as electromagnetic stimulation), and intelligent biomaterial-based delivery systems, to modulate eNSCs behavior and reconstruct a regenerative microenvironment synergistically. In parallel, applying advanced technologies such as high-throughput single-cell sequencing, spatial transcriptomics, and lineage tracing will be instrumental in elucidating the fate-determining mechanisms of distinct eNSCs subpopulations and providing theoretical support for precision interventions. Furthermore, the establishment of large animal SCI models that more closely mimic human pathological conditions, along with the development of three-dimensional organoid systems and biomimetic microenvironments, is expected to enhance the clinical translatability of experimental outcomes substantially. Systematic optimization of treatment windows, delivery doses, and safety profiles will also ensure a smooth transition from bench to bedside.

In conclusion, with the continuous integration of tissue engineering, biomaterials, artificial intelligence, and neuromodulation technologies, the construction of multifunctional scaffold systems featuring controllable release profiles and strong cell compatibility holds great promise for enhancing neuronal differentiation of eNSCs, reconstructing central neural networks, and ultimately driving a shift in SCI treatment from structural repair to functional restoration. A multidisciplinary approach is anticipated to provide more clinically translatable regenerative solutions for spinal cord injury patients and promote both the standardization and personalization of neuroregenerative therapies.
